# Identification of monotonically differentially expressed genes for non-small cell lung cancer

**DOI:** 10.1186/s12859-019-2775-8

**Published:** 2019-04-11

**Authors:** Suyan Tian

**Affiliations:** Division of Clinical Research, The First Hospital of Jilin University, 71 Xinmin Street, Changchun, 130021 Jilin China

**Keywords:** Non-small cell lung cancer (NSCLC), Monotonically expressed genes (MEGs), Pathologic stages, Overall survival, Feature selection, Adenocarcinoma, Squamous cell carcinoma

## Abstract

**Background:**

Monotonically expressed genes (MEGs) are genes whose expression values increase or decrease monotonically as a disease advances or time proceeds. Non-small cell lung cancer (NSCLC) is a multistage progression process resulting from genetic sequences mutations, the identification of MEGs for NSCLC is important.

**Results:**

With the aid of a feature selection algorithm capable of identifying MEGs – the MFSelector method – two sets of potential MEGs were selected in this study: the MEGs across the different pathologic stages and the MEGs across the risk levels of death for the NSCLC patients at early stages. For the lung adenocarcinoma (AC) subtypes no statistically significant MEGs were identified across pathologic stages, however dozens of MEGs were identified across the risk levels of death. By contrast, for the squamous cell lung carcinoma (SCC) there were no statistically significant MEGs as either stage or risk level advanced.

**Conclusions:**

The pathologic stage of non-small cell lung cancer patients at early stages has no prognostic value, making the identification of prognostic gene signatures for them more meaningful and highly desirable.

## Background

The 5-year survival rate of non-small cell lung cancer (NSCLC), which accounts for approximately 85% of lung cancer (LC) cases, remains very low [1]. So far, the most promising strategy of improving progression-free survival time and overall survival (OS) time of the NSCLC patients is early diagnosis followed by surgical resection, which is currently the standard of care. Unfortunately, at the time of diagnosis most NSCLC patients have already progressed to the advanced or metastatic stages, which are inoperable [2].

Since NSCLC is a multistage progression process resulting from genetic sequences mutations, researchers may be interested in knowing how the expression pattern of a gene varies as NSCLC progresses from early to late stages. This is especially the case with “monotonic” genes whose expression levels increase or decrease monotonically as the disease advances. Such an investigation may harvest no meaningful results given that no satisfactory segmentations among the early stages of NSCLC using gene expression profiles have yet been achieved [3–5]. Therefore, the likelihood of finding monotonically expressed genes (MEGs) across pathologic stages is extremely low.

Survival rates for stages I through IV of NSCLC decrease due to the progress of the disease such as the 5-year survival rate for stage I is 47%, stage II is 30%, stage III is 10%, and stage IV is 1% (http://www.cancer.org). These pathologic stages were determined according to the Cancer Staging System (http://cancerstaging.org) of the American Joint Committee on Cancer (AJCC). Nevertheless, for the NSCLC patients at the early stages of disease, the pathologic stage may not be a good index of how long a patient can expect to be progression-free and survive, given that a study by Der et al. [6] showed the hazard ratio for stage II versus stage I was 1.52 (95% CIs: 0.9~2.55) and the corresponding *p*-value of the log-rank test which compared the survival curves of patients at stage I and stage II was 0.11. Meanwhile, continuous efforts [7–13] have been made to identify attributes predictive of progression-free survival or overall survival (mainly using gene expression profiles, thus an attribute corresponds to a gene), which may facilitate personalized medicine. Only with more “personalized” treatments that are tailored for a specific patient, could a stage I patient with poor prognosis live longer by receiving the adjuvant chemotherapy while a stage II patient with good prognosis could avoid suffering from adverse effects associated with the treatments and have a better quality of life.

Among NSCLC patients, adenocarcinoma (AC) and squamous cell carcinoma (SCC) are two major subtypes, accounting for roughly 40 and 35% of the lung cancer (LC) cases, respectively. Increasing evidence supports the fact that AC and SCC differ in many respects [14], therefore AC and SCC have been regarded as two distinct diseases. The DEGs for the two subtypes versus the normal controls naturally vary from each other. Hence, it is quite possible that MEGs across multiple pathologic stages for AC and SCC are distinct. Separate analyses for each subtype to identify their respective prognostic genes and respective MEGs are more appropriate than the analyses where these two subtypes are considered together as a whole.

In this study, a novel feature selection algorithm capable of identifying monotonically changed genes i.e., the monotonic feature selector (MFSelector) method [15] was used to test two research hypotheses. In [15], the authors demonstrated that the MFSelector method outperforms other competitive methods capable of identifying MEGs. The first hypothesis of the current study is to test if MEGs exist across different pathologic stages (i.e., stages IA, IB, IIA and IIB). The second hypothesis is to test the existence of MEGs over different risk profiles of a disease. Here, the risk levels of death were determined on the basis of the quartiles of overall survival time, namely, the extremely high-risk group whose survival time is less than 25% of survival time of all samples, the high-risk group is in between 25 and 50%, the moderate-risk group is in between 50 and 75% and the low-risk group is above 75%. My conjecture is that there are no MEGs across pathologic stages but some MEGs do exist over different risk levels. If this conjecture is true, it is more beneficial to predict a patient’s risk of death using his/her gene expression profiles than using his/her pathologic stage alone, which provides more support for the necessity of finding genes with significant prognostic values for NSCLC patients at early stages.

## Methods

### Experimental data

The raw data of these two microarray experiments are publicly accessible on the GEO (https://www.ncbi.nlm.nih.gov/geo/) repository, under the accession numbers of GSE37745 [16] and GSE50081 [6]. All chips of these two experiments were profiled on the Affymetrix HGU133 Plus 2.0 platform. Only patients in these two cohorts who remained adjuvant treatment naïve with their clinical information such as survival time, age and smoking status available were included. Furthermore, in order to avoid ambiguous classification of a patient into a risk group (corresponding to the four quartiles of overall survival time), all censored patients were excluded from the downstream analyses, resulting in 73 AC patients and 31 SCC patients included in this study. Among the 73 AC patients, there were 11 stage IA, 39 stage IB, 5 stage IIA and 18 stage IIB patients. For SCC patients, there were 6 stage IA, 18 stage IB, 0 stage IIA and 7 stage IIB.

RNA-Seq data were downloaded from The Cancer Genome Atlas Data Portal (level 3) (https://tcga-data.nci.nih.gov/tcga/). Cohorts that were considered are: LUAD for the AC subtype and LUSC for the SCC subtype. By restricting selection to patients at early stages of disease and whose vital status was decreased, 73 AC and 97 SCC patients were included in this analysis. Among the 73 AC patients, there were 14 stage IA, 26 stage IB, 8 stage IIA and 23 stage IIB. For SCC patients, there were 17 stage IA, 46 stage IB, 12 stage IIA and 22 stage IIB. Notably, the adjuvant treatment restriction has been released for the RNA-Seq data since with it, there were only 17 AC and 9 SCC patients left.

### Pre-processing procedures

Raw data (CEL files) of these two microarray datasets were downloaded from the GEO repository. The expression values were obtained using the fRMA algorithm [17] and were normalized using quantile normalization. Then, the COMBAT algorithm [18] was used to eliminate or alleviate the batch effects existing between these two experiments. Counts-per-million (CPM) values for the RNA-seq data were calculated and log2 transformed by the Voom function in the R software.

### Monotonic feature selector

The monotonic feature selector (MGSelector) method proposed by Wang et al. [15] introduces a new novel index, namely, the DE_total_ (total discriminating error) score for each gene, with the objective of selecting genes with strong monotonically changed patterns over stages or time. In addition, the corresponding *p*-value and q-value (which adjusts for the multiple comparisons) of this score was calculated using permutation tests to determine the significance level of a gene. There are two sets of monotonically expressed genes, namely a monotonically increasing/ascending set and the monotonically decreasing/descending set. The MGSelector method was described briefly as below using the ascending set as an example.

Suppose there are K levels/time points, it is natural to expect subjects in the lower levels have smaller expression values compared to the subjects in the remaining levels for a monotonically increasing expressed gene. First, n_1_ (here, n_1_ is the number of patients at the first level) discriminating lines may be drawn over the expression value of each patient at the first level. The samples at level I above this line and the patients at other levels below this line were misclassified into their opposite classes, and the number of misclassified samples (i.e., “discriminating errors”) was counted. The final discriminating line to differentiate the level I from the other levels corresponded to the line with the least number of discriminating errors. This step was repeated for K-1 times to discriminate the patients at the first k (k = 1,2, … K-1) levels apart from the remaining patients, resulting in K-1 discriminating lines. If a gene has K-1 distinct discriminating lines and the lines for a higher level are above the lines for a lower level, the expression change pattern of this specific gene presents a perfect monotonically increasing expression tendency. For the monotonically decreasing expression pattern, the discriminating lines taking the reversed orders with the line for a higher level below the line for a lower level were made.

**Fig. 1:**
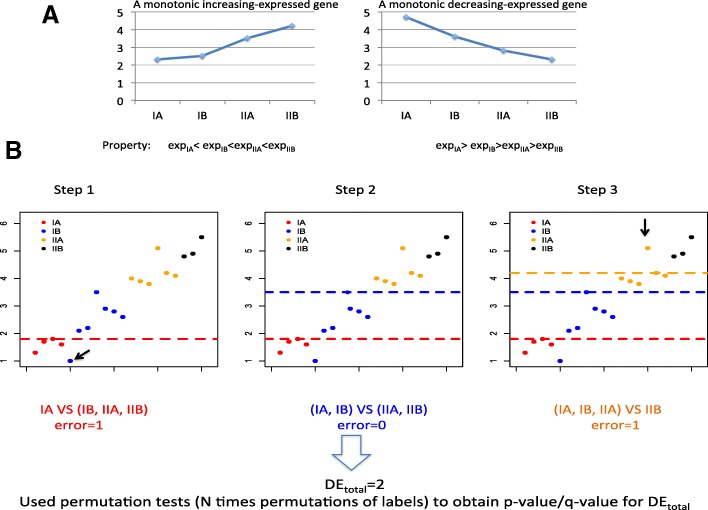
Graphical illustrations on what a MEG is and the MFSelector method. **a** The definition for a MEG. **b** The flowchart of the MFSelector method

The DE_total_ score is the sum of discriminating errors for all these K-1 segmentations. Given the DE_total_ score does not make any distributional assumption on the data, it is less sensitive to the biases caused by outliers. Furthermore, a *p*-value and/or a q-value of the DE_total_ score were calculated using permutation tests to determine if a gene was a statistically significant MEG or not. Figure 1 provides a graphical illustration for the definition of a MEG and the flowchart of the MFSelector method.

### Biological relevance and gene set analysis

The biological relevance of the identified AC-specific MEGs and SCC-specific MEGs was investigated by searching the GeneCards database [19]. Then, the gene set enrichment analysis were carried out using the String software [20].

### Statistical language and packages

All statistical analyses were carried out in the R language version 3.3.3 (www.r-project.org), and the R codes of the MFSelector algorithm were downloaded from the following webpage (http://microarray.ym.edu.tw/tools/MFSelector). Additionally, the Venn-diagrams were made with the aid of an online bioinformatics tool (http://bioinformatics.psb.ugent.be/webtools/Venn/).

## Results

### Integrated microarray data

#### MEGs across pathologic stages

Using the integrated microarray data and the MFSelector method, the MEGs across pathologic stages for the AC and SCC subtypes were separately identified. First, the AC patients and SCC patients were rearranged in an ascending order according to their pathologic stages, respectively.

The top-ranked genes are listed separately for the AC and SCC subtypes in Table 1, and then within each subtype the genes are listed separately for the ascending expressed pattern and the descending expressed pattern. From this table, it was observed that if the cutoff of the corresponding *p*-value was set at 0.05, there were no statistically significant MEGs for AC and SCC subtypes. If a less stringent threshold of 0.1 was chosen, only 18 genes in the AC ascending category were deemed to be statistically significant. As expected, no MEGs across pathologic stages can be identified if the cutoff for *p*-values/q-values is set at 0.05, a predominant default value used in the field of statistics.

**Table 1:** The top-ranked genes across the pathologic stages by the MFSelector method

AC		SCC	
Ascending	Descending	Ascending	Descending
DGCR14 ^*^	AIMP1	GRHL3	CD97
SART1 ^*^	CXADR	KIAA0907	DUSP23
SLC34A1 ^*^	SOCS6	TM4SF1	FBXW7
TYR ^*^	TM2D1	AKAP7	GKN2
CDH4 ^*^	ZBTB43	CFL1	PAG1
CNTROB ^*^	ALCAM	DBF4B	PAX5
CRYBA1 ^*^	ATP5L	F11R	RILPL2
GCK ^*^	DYNC2H1	PHF12	SFTPB
L1CAM ^*^	PARD6B	PIP5K1A	TNNC1
LILRA3 ^*^	POLI	RGS7	TSPAN1
MATN4 ^*^	RAB22A	RPL8	
NPAS4 ^*^	RND3	TFAP2A	
NR4A3 ^*^	RPS21		
SCNN1A ^*^	SCP2		
SLC39A2 ^*^	UBE2E1		
SMARCD3 ^*^			
STMN4 ^*^			
TPSAB1 ^*^			

Note: * the corresponding q-value of DE_total_ < 0.1; ** q-value< 0.05; AC: lung adenocarcinoma; SCC: lung squamous cell carcinoma; Ascending: a monotonically expressed gene in the increasing order; descending: a monotonically expressed gene in the decreasing order

#### MEGs across risk levels of death

For this objective, the MFSelector method was used to identify MEGs across risk strata separately for the AC and SCC subtypes. Using the first quartile, the median and the third quartile of overall survival time for those 73 AC patients as cutoffs, AC patients were divided into four groups with approximately 18 patients in each category. Likewise, SCC patients were also categorized into four groups with the number of patients in each group being approximately eight.

The most significant genes (with the least q-values) in this analysis are presented in Table 2. From this table, it was observed that for the AC subtype, 74 genes (48 were ascending expressed and 26 were descending expressed) were identified as the MEGs even with the more stringent cutoff (i.e., 0.05 for the q-value) being chosen. Nevertheless, for the SCC subtype, no genes were met with the less stringent cutoff of 0.1, let alone 0.05. The following two reasons may explain why no statistically significant MEGs were identified for this subtype. One is that the sample size of SCC patients in the experimental data was not big enough to have statistically significant results. The other possible explanation is the evolution of this disease is not a process of quantitative accumulation, implying the non-existence of MEGs indeed for the SCC subtype. Further investigation is warranted.

**Table 2:** The top-ranked genes across the risk levels of death by the MFSelector method

AC				SCC	
Ascending		Descending		Ascending	Descending
GNL3L^**^	FKBP3 ^**^	RGS2 ^**^	ARF6^a^	MMP1	ATIC
RALY^**^	FRAT1 ^**^	POLK^**^	C21orf91^a^	S100A2	SART3
TBC1D10A^**^	GRK7^**^	SHOC2 ^**^	ELK3 ^a^	TP53AIP1	CRLS1
CDK19 ^**^	HINFP ^**^	ADD3 ^**^	EPS15^a^	DEFB1	DST
CRY2 ^**^	INPP5K ^**^	EXOC1 ^**^	IBTK^a^	EIF2S1	EMG1
EPHB2 ^**^	KLF13 ^**^	KRAS ^**^	KPNA5^a^	HSPG2	FES
MARK4 ^**^	MC4R ^**^	SLC4A1AP^**^	MTF2 ^a^	IBTK	HSP90AB1
NAT6 ^**^	MDH2 ^**^	DMTF1^**^	MYL12A^a^	PAK2	PEBP1
PLEKHJ1 ^**^	MKNK2 ^**^	FRYL^**^	PPP1CB^a^	SH2D3C	PMS1
RAB1B ^**^	NARF ^**^	ITGAV^**^	RUFY2^a^	SPHK1	PPP6C
RHOT2 ^**^	PACSIN2 ^**^	KIF5B^**^	RWDD1^a^	UBR7	
TMEM222 ^**^	PRKACA ^**^	SOD2^**^	STX7^a^		
ANP32A ^**^	RARA ^**^	USO1^**^	TBK1^a^		
CORO1B ^**^	RFXANK ^**^				
FNTB ^**^	SIRT2 ^**^				
MAP 7D1 ^**^	STAT3 ^**^				
PI4KA ^**^	TACR2 ^**^				
SHBG ^**^	TMEM39B^**^				
THRA ^**^	TPPP ^**^				
TINF2 ^**^	TSSC4 ^**^				
UPF1 ^**^	TUFM ^**^				
ZNF142 ^**^	URGCP ^**^				
DLG5 ^**^	WASF2 ^**^				
EP400 ^**^	YIF1A ^**^				

Note: * the corresponding q-value of DE_total_ < 0.1; ** q-value< 0.05; ^a^ q-value = 0.05; AC: lung adenocarcinoma; SCC: lung squamous cell carcinoma; Ascending: a monotonically expressed gene in the ascending order; descending: a monotonically expressed gene in the descending order. If the cutoff of q-value was set at 0.1, there were 166 ascending MEGs and 172 descending MEGs for the AC subtype. However, no MEGs exist for the SCC subtype at the level of 0.1

**Fig. 2:**
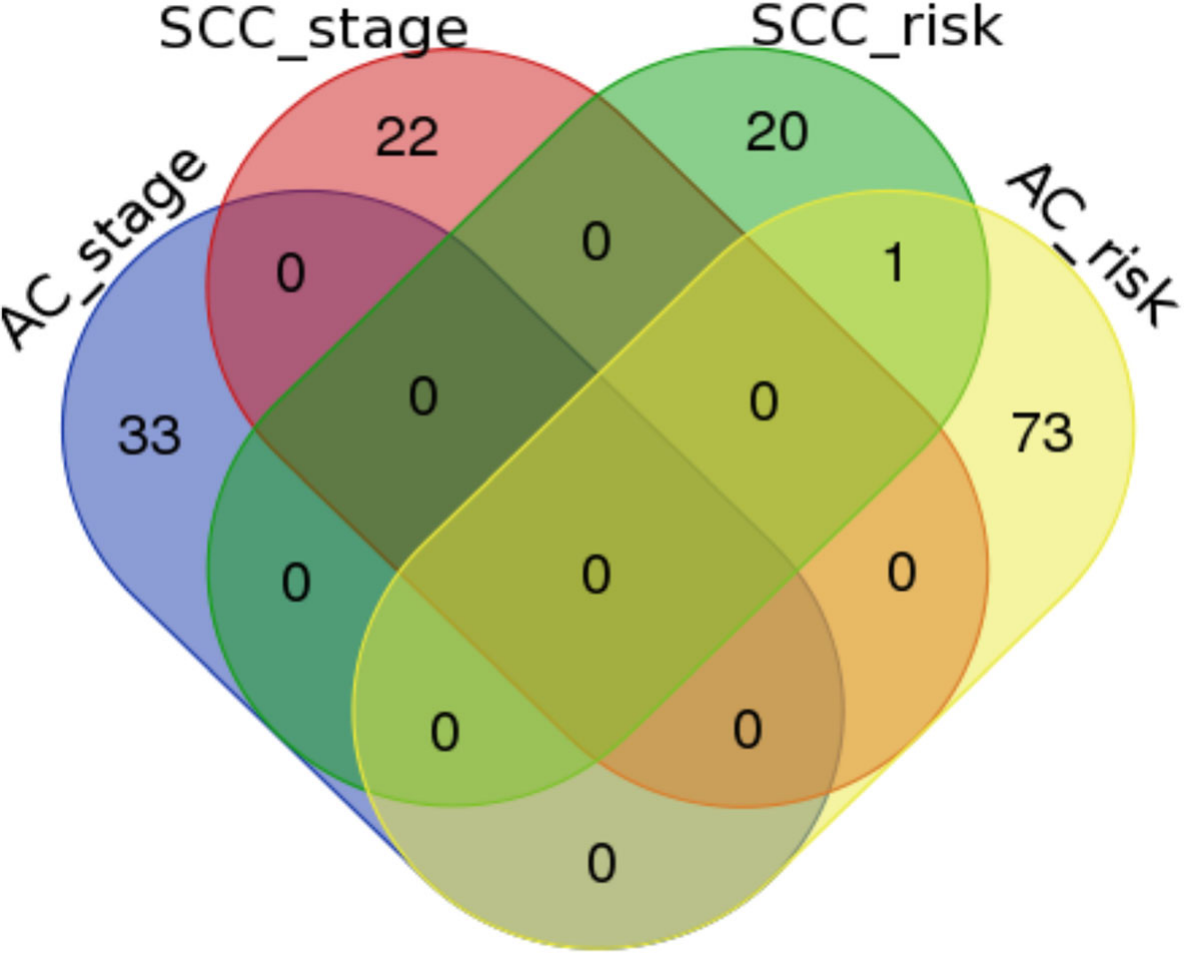
Venn-diagram of the top-ranked genes among the four categories based on subtype and risk status/stage. AC_stage: across stages for lung adenocarcinoma subtype; SCC_stage: across stages for lung squamous cell carcinoma subtype; AC_risk: across risk levels of death for lung adenocarcinoma subtype; SCC_stage: across risk levels of death for lung squamous cell carcinoma subtype. There is only one overlapped gene between the SCC_risk and the AC_risk categories, i.e., IBTK that is indirectly related to lung cancer due to its association with KRAS, AKT1, BRAF and MAPK1 according to the GeneCards database

A Venn-diagram among these four categories is given in Fig. 2. There was only one overlapped gene, i.e., IBTK between the SCC & risk and AC & risk categories. The lack of overlapping between these two categories may imply two things. The first implication is that the identified MEGs are indeed subtype-specific. Therefore, either separate analyses for the AC and SCC subtypes or the utilization of statistical methods capable of identifying subtype-specific genes is highly recommended. The other is that the histological stage may not be a good representation of the risk status for overall mortality.

Besides the pathologic stage and the risk level of death, one clinical variable, i.e., a patient’s age was considered. Similarly, using the first quartile, the median and the third quartile of age as cutoffs, both AC patients SCC patients were divided into four approximate equal-sized categories. For this index, no significant MEGs were identified for either AC subtype or SCC subtype, even at the less stringent cutoff for *p*-value of 0.1.

### RNA-seq data

Focusing on the pathologic stages and the risk levels of being death, the MFSelector method was applied to the RNA-seq data to find the MEGs. Then, a comparison between the MEGs identified using microarray data and the MEGs identified using RNA-seq data was made. Unfortunately, there were no statistically significant MEGs identified for all four categories— the AC & stage category, the AC & risk category, the SCC & stage category and the SCC & risk category at the level of 0.1. Nevertheless, the top-ranked genes in the risk categories tended to have smaller *p*-values than those in the stage categories. For example, the corresponding *p*-value of the first-ranked descending gene for the SCC & stage category was 0.149 whereas that of the descending gene on the top of the SCC & risk category was only 0.116.

Additionally, two other potential indices of the disease advancement were considered, namely, the number of packs of cigarettes smoked per year and the age at which the disease was diagnosed. Of note, such information is not available for the integrated microarray dataset. For both of these indices, no significant MEGs were identified for either AC or SCC at the level of 0.1.

Since the RNA-seq data included more hetero-generous patients in terms of adjuvant treatment option (including those that had adjuvant treatments and the status was unknown, in addition to those who remained adjuvant treatments naïve), it is unsurprising to observe the inconsistent results between the RNA-seq data and the microarray data.

## Discussion

The biological relevance of those top-ranked genes listed on Tables 1 and 2 was searched in the GeneCards database. Focusing on the direct association supported by the literature, the searching results elucidated that for the AC & stage category, only ALCAM, TYR and SART1 are related to lung adenocarcinoma but with very low confidence scores (1.60, 0.25 and 0.25 respectively). By contrast, for the AC & risk category KRAS, STAT3, SOD2, KIF5B, ITGAV, EPHB2, RALY, ARF6, TBC1D10S, UPF1 and RARA are indicated to be directly related to lung adenocarcinoma. Among them, KRAS has the highest confidence score. It is well known that KRAS mutations are the most frequently oncogene aberrations in NSCLC patients [21, 22]. In this study, the identification of KRAS as a significant MEG points out a new direction to view the roles it may play during AC progression, from the perspective of expression values instead of somatic mutations. Likewise, STAT3 has a very high confidence score and many articles in the literature support its association with AC. For instance, a study by Qu et al. [23] stated that “… in both lung cancer and chronic obstructive pulmonary disease, the STAT3 gene was up-regulated” and “… STAT3 and its downstream genes can serve as biomarkers for lung adenocarcinoma …” . Therefore, many top-ranked genes in this category not only are statistically significant but also have meaningful biological interpretation.

For the SCC & stage category, none of the top-ranked genes were found to correlate with SCC directly. On the other hand, three genes, i.e., PEBP1, S100A2 and HSP90AB1, are directly related to SCC for the SCC & risk status category according to the GeneCards database. Even though the top-ranked genes identified in both categories are not statistically significant, the genes in the risk category have apparently better biological relevance. This is in consistent with the results for AC, implying that histological stage may not be a good representative for the risk of death and thus may only have a limited prognostic value for the NSCLC patients at early stages.

**Fig. 3:**
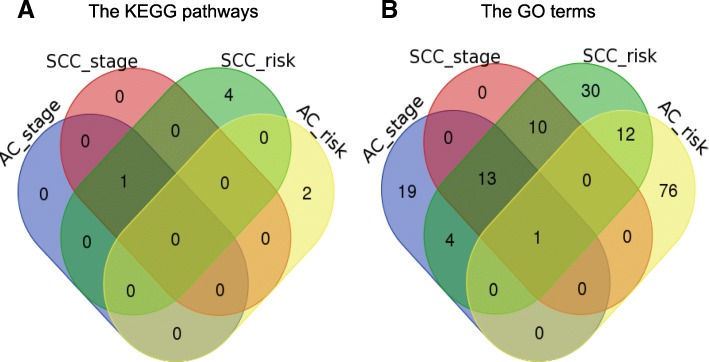
Venn-diagram of the enriched KEGG pathways and GO terms between the four categories based on subtype and risk status/stage. **a** The enriched KEGG pathways; **b** The GO terms. Overall, the consistency level at the pathway level is higher. For the KEGG pathways, the ribosome pathway was commonly enriched in the AC_stage, SCC_stage and SCC_risk categories. For the GO terms, there is one term enriched by all four categories, 13 terms by all categories but the AC_risk category, 4 terms by the AC_stage and SCC_risk categories and 12 terms by the AC_risk and SCC_risk categories. AC_stage: across stages for lung adenocarcinoma subtype; SCC_stage: across stages for lung squamous cell carcinoma subtype; AC_risk: across risk levels of death for lung adenocarcinoma subtype; SCC_stage: across risk levels of death for lung squamous cell carcinoma subtype

As far as the enriched pathways were concerned, the degree of stability increased. The String software (www.string-db.org) indicated 89, 37, 81 and 24 GO terms were enriched by the identified genes for the AC & risk category, the AC & stage category, the SCC & risk category and SCC & stage category, respectively. Among 165 unique enriched GO terms, only one term was commonly enriched by all four categories. Moreover, 13 terms were enriched by three categories and 14 terms were enriched by two categories, summing up to 40 overlapped terms. The 40 (40/165 ≅ 24.2%) overlapped GO terms are listed in Table 3. For the KEGG pathways, there were 2 enriched pathways for the AC & risk category, 1 for AC & stage, 5 for SCC & risk and 1 for SCC & stage. Only one overlapped pathway (1/7 ≅ 14.3%), the ribosome pathway, was commonly enriched in the AC & stage, SCC & stage and SCC & risk categories. The Venn-diagrams of the KEGG pathways and the GO terms enriched in these four categories are given in Fig. 3. Although the overlapping proportion showed some increment compared to that at the gene level, it was still not substantially large. Therefore, the subtype-specific-gene conclusion remains true at the pathway level, namely the enriched pathways tend to be unique for AC and SCC subtypes.

**Table 3:** The overlapped GO terms

Category	GO terms
All	Cytosol
All but AC_risk	translational termination
	translational elongation
	translational initiation
	nuclear-transcribed mRNA catabolic process, nonsense-mediated decay
	SRP-dependent cotranslational protein targeting to membrane
	viral life cycle
	viral transcription
	cellular protein complex disassembly
	viral gene expression
	cytosolic ribosome
	cellular macromolecule catabolic process
	protein localization to membrane
	focal adhesion
AC_stage & SCC_risk	structural constituent of ribosome
	cellular component disassembly
	mRNA metabolic process
	Translation
SCC_risk & SCC_stage	establishment of protein localization to membrane
	viral process
	eyelid development in camera-type eye
	large ribosomal subunit
	cytosolic large ribosomal subunit
	establishment of protein localization to plasma membrane
	ribosome
	intracellular protein transport
	multi-organism process
	protein targeting to membrane
AC_risk & SCC_risk	enzyme binding
	purine ribonucleoside triphosphate binding
	single-organism intracellular transport
	purine ribonucleotide binding
	single-organism transport
	cytoplasmic transport
	localization
	kinase binding
	purine ribonucleoside binding
	nucleotide binding
	cellular protein metabolic process
	cellular protein localization

Note: AC_stage represents across stages for lung adenocarcinoma subtype; SCC_stage represents across stages for lung squamous cell carcinoma subtype; AC_risk represents across risk levels of overall mortality for lung adenocarcinoma subtype; SCC_stage represents across risk levels of overall mortality for lung squamous cell carcinoma subtype

**Fig. 4:**
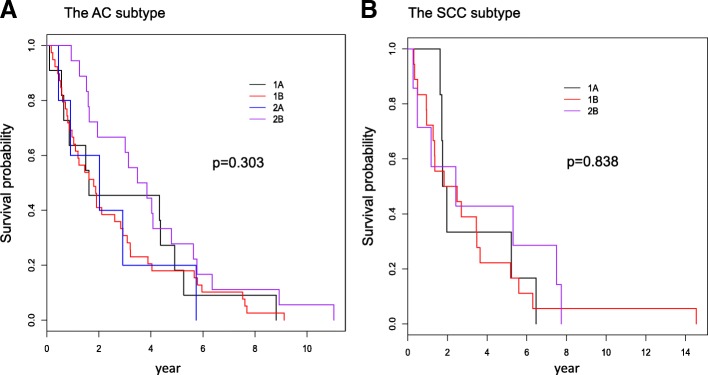
Kaplan-Meier plots for the overall survival of non-small cell lung cancer stratified by the pathologic stages. **a** The AC subtype; **b** The SCC subtype. Here, p stands for the *p*-values of corresponding log-rank tests. AC: lung adenocarcinoma; SCC: lung squamous cell carcinoma

To further explore whether the pathologic stage may serve as a surrogate for the risk of death, the Spearman’s correlation coefficient between the risk level and the pathologic stage of patients was calculated, and Kaplan-Meier plots were made to compare the survival curves of different pathologic stages and log-rank tests were carried out to determine if the differences between those survival curves were statistically significant. The Spearman’s correlation coefficient for the AC subtype was estimated as 0.19 with a *p*-value of 0.11, and for the SCC subtype it was 0.02 with a p-value of 0.91. Figure 4 presents the Kaplan-Meier plots, the p-value of the log-rank test for AC was 0.303, and for SCC it was 0.838. Based on these tests, it was concluded that there is no significant association between pathologic stage and risk of death for the NSCLC patients at early stages.

## Conclusions

With the aid of a feature selection algorithm capable of identifying MEGs, namely the MFSelector method [15], two research hypotheses were investigated. Using an integrated microarray dataset, the analyses showed that across pathologic stages there were no statistically significant MEGs whereas dozens of MEGs across risk levels of death were identified for the AC subtype. For the SCC subtype, however, there were no statistically significant MEGs in either stage or risk level categories. This may be explained by that either the sample size of the SCC subtype in this study is too small to guarantee an adequate statistical power or the disease progression of SCC differs.

The results of this study suggest that the pathologic stage is not well correlated with the risk of death and thus necessitate that the construction of prognostic gene signatures for NSCLC patients. More work is especially needed to develop more machine learning models capable of selecting subtype-specific prognostic genes readily and to boost the real-world applications of those models.

## Abbreviations

AC: Lung adenocarcinoma
CPM: Counts-per-million
GEO: Gene Expression Omnibus
GO: Gene ontology
KEGG: Kyoto Encyclopedia of Genes and Genomes
MEG: Monotonically expressed gene
MFSelector: Monotonic feature selector
NSCLC: Non-small cell lung cancer
OS: Overall survival
SCC: Squamous cell lung carcinoma
